# Recognition of Hydrophilic Cyclic Compounds by a Water-Soluble Cavitand

**DOI:** 10.3390/molecules26071922

**Published:** 2021-03-30

**Authors:** Yun-Hui Wan, Yu-Jie Zhu, Julius Rebek, Yang Yu

**Affiliations:** 1 Center for Supramolecular Chemistry & Catalysis and Department of Chemistry, College of Science, Shanghai University, 99 Shang-Da Road, Shanghai 200444, China; wanyunhui@shu.edu.cn (Y.-H.W.); zyj40008764@shu.edu.cn (Y.-J.Z.); 2 Skaggs Institute for Chemical Biology and Department of Chemistry, The Scripps Research Institute, 10550 North Torrey Pines Road, La Jolla, CA 92037, USA; jrebek@scripps.edu

**Keywords:** water-soluble cavitand, hydrophilic molecule recognition, hydrogen bond, hydrophobic effect

## Abstract

A water-soluble deep cavitand bearing amides on the upper rim and trimethyl ammonium groups on the feet was synthesized. The open-ended cavity is stabilized by the intramolecular hydrogen bonds formed between the adjacent amides, and the introduction of trimethylammonium imparts to the cavitand good solubility in water. The cavitand exhibits high binding affinity and selectivity to hydrophilic molecules in water. With certain guests, such as cyclohexyl alcohols, amines and acids, the recognition involves the synergistic action of hydrogen bonding with hydrophobic effects. The binding phenomena are interpreted in terms of a fixed solvent cage presented by the host to the guest.

## 1. Introduction

Molecular recognition [1] is a central topic of supramolecular chemistry and provides a basis for numerous life processes [2], such as signal transduction, cell recognition and so on. Synthetic receptors are powerful tools for supramolecular chemists to mimic the complex biomolecular recognition process in a laboratory. In the past few decades, a number of synthetic molecular receptors, such as crown ethers [3], cyclodextrins [4], calixarenes [5], cucurbiturils [6] and pillararenes [7], have been synthesized and extensively studied. Their readily accessible macrocyclic structures and promising applications in molecular recognition have attracted synthetic, physical organic and supramolecular chemists. Recognition of ions and hydrophobic molecules in aqueous medium has been widely reported [8,9,10,11], but the recognition of neutral hydrophilic molecules in water remains a challenging task [12,13]. The effective solvation of hydrophilic molecules thwarts their attraction to synthetic receptors since the recognition process involves a desolvation penalty that needs to be compensated. Natural receptors solve this problem cleverly by combining hydrophobic effects with well-positioned non-covalent interactions, such as hydrogen bonding. For example, lectins—the bioreceptors of carbohydrates—can selectively recognize highly hydroxylic carbohydrate molecules by decorating their hydrophobic cavities with hydrogen bonding sites [14]. Supramolecular chemists have designed such inwardly directed functions to imitate such biomimetic receptors, but few examples have been successful [15,16].

Cavitands are macrocyclic hosts, members of the family derived from resorcinarenes. Since their introduction by Cram and co-workers [17] in the early 1980s, cavitands have drawn supramolecular chemists’ attention and interest, and have grown rapidly during the past decade. With a hydrophobic cavity and one open end [18], cavitands can serve as synthetic containers for molecular recognition [19], sequestration of physiologically relevant molecules [20] and even facilitating reactions as vessels and catalysts [21]. Typically, cavitands have been extensively studied in organic medium, but now chemists are focusing their attention on cavitands in water. Why water? Water is ubiquitous and is friendlier to the ecosystem than petrochemical-derived solvents. Water provides an environment for life, and participates in and mediates numerous biological processes [22]. Application of cavitands to mimic natural receptors requires that these synthetic hosts are soluble in water. Here, we apply peripheral amides and pendant trimethyl ammonium groups to develop a cavitand with high solubility and characterize its recognition of guests in water.

## 2. Results

**Figure 1:**
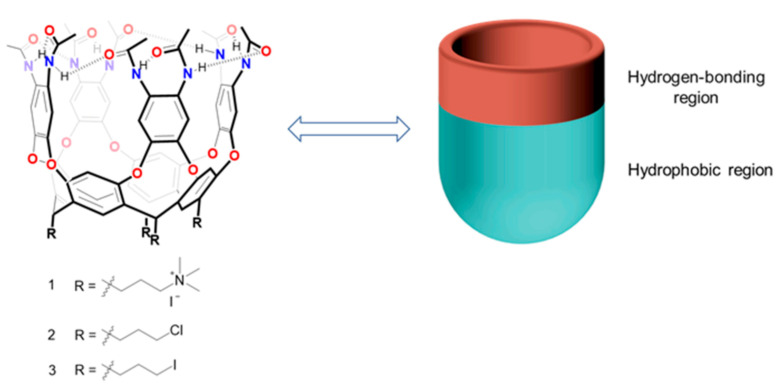
Chemical structures of the water-soluble cavitand **1** and its precursors. The cartoon highlights the environments inside the cavity.

The synthesis of self-folding cavitands was reported previously, and its “self-folding” name came from the intramolecular hydrogen bonds that maintain the vase shape as opposed to the kite shape which features larger exposure to the solvents [17]. Its ability to bind small molecules was extensively studied in organic solvents [23], but water-soluble versions, bearing ionizable, carbohydrate or oligoethylene glycol feet, were harder to prepare. The target cavitand **1** (Figure 1) was intended to shorten the process and provide a cavitand that would dissolve in water over a wide pH range.

**Scheme 1:**
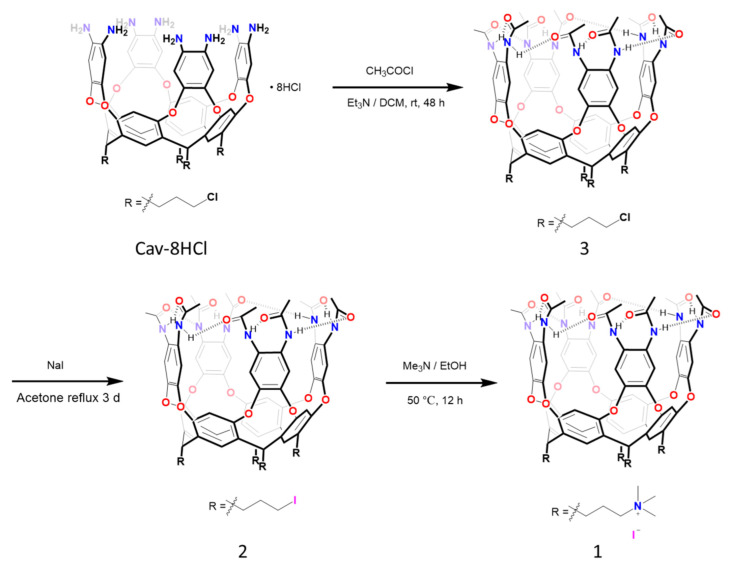
Route for the synthesis of water-soluble cavitand **1.**

The octa-amino cavitand was prepared as reported before [24] and acetylation with acetyl chloride in dichloromethane at room temperature gave the octa-amide cavitand **2** with chloride feet in good yield (Scheme 1). Cavitand **2** was then refluxed with excess of sodium iodide in acetone for 3 days to afford **3** with tera-iodine in an excellent isolated yield. Cavitand **3** was further treated with trimethyl amine in ethanol solution, giving **1** as a light yellow solid (90% yield). The four-trimethyl ammonium feet of **1** permit good solubility in water to give a colorless solution. All the precursors and water-soluble cavitand **1** were characterized by ^1^H NMR, ^13^C NMR and high-resolution electrospray ionization mass spectroscopy (Figures S1 and S11).

In apolar solvents the octa-amide cavitand has a vase conformation stabilized through the formation of intramolecular hydrogen bonds: the amide-amide C = O H−N interactions between adjacent benzenes and on the same benzene create a seam of eight hydrogen bonds in a circle, stitched along the upper rim in a head-to-tail manner, in a clockwise or counterclockwise arrangement [25]. The hydrogen bond seam resists the rotation of amides and the adjacent amide N-H protons experience a different magnetic environment. The N-H resonances appear as two signals at 10.0 and 9.2 ppm in the ^1^H NMR spectrum of **3** in chloroform-*d* (Figure S3). However, polar solvents such as dimethylsulfoxide can act as competitive hydrogen bond acceptors. The hydrogen bond cycle collapses in a CDCl_3_-DMSO-*d*_6_ (vol/vol = 9:1) mixture, which can be deduced from the ^1^H NMR spectrum. In this mixture, the amide N-H protons appear as one singlet at 9.57 ppm (Figure S4).

Like other dynamic cavitands [26,27], **1** displays interconversion between receptive “vase” and unreceptive “kite” conformations in solution. The vase form shows a characteristic methine signal around 5.5 ppm in the ^1^H NMR spectrum, while the kite form features the methine signal at about 4.0 ppm. The ^1^H NMR spectra of cavitand **1** was taken in a different solvent such as DMSO and water. In DMSO the chemical shift of the methine peak was observed at 5.67 ppm, which shows an exclusive vase conformation of **1** with DMSO as guest inside. In D_2_O the methine shifted to upfield at 4.12 ppm, revealing that the **1** exists in a kite conformation. In the absence of a suitable guest, the cavitand only takes a kite form and, in order to minimize the exposure of the hydrophobic interior surface to polar solvents, the kite prefers to dimerize through stacking of aromatic panels into a velcrand. In the presence of appropriate guests that can solvate the concave interior of cavity, a shift in equilibrium to the vase conformation is achieved.

**Figure 2:**
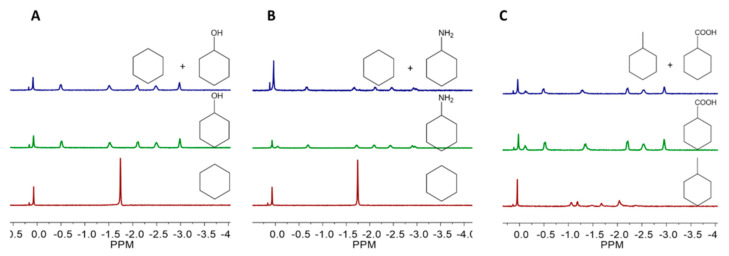
Partial comparative ^1^H NMR spectra of cavitand **1** upon adding from bottom to top (**A**): cyclohexane, cyclohexanol, mixture of cyclohexane and cyclohexanol, (**B**): cyclohexane, cyclohexylamine, mixture of cyclohexane and cyclohexylamine, (**C**): methylcyclohexane, cyclohexanecarboxylic acid, mixture of methylcyclohexane and cyclohexanecarboxylic acid.

We explored the binding ability of **1** using various guests in aqueous solution. Since the hydrophobic effect is a main driving force for binding organic molecules in water, we looked first at cyclic hydrocarbons as fits for the hydrophobic cavity of **1**. The chemical shifts of such bound guests typically appear in the upfield region (below 0 ppm) in the ^1^H NMR spectra, as a consequence of shielding by the 8 aromatic panels of the host. On brief sonification with **1** in aqueous solution, both cyclohexane and methylcyclohexane showed such signals, and the integration indicated 1:1 (stoichiometric) host–guest complex formation. The bound cyclohexane shows only one peak at −1.8 ppm (Figure 2A and Figure S12), indicating all the protons experienced an identical shielding effect. It also indicates that cyclohexane is tumbling and rotating rapidly in the cavity on the NMR time scale. As for methylcyclohexane, the bound guest shows a full set of peaks in negative region in the ^1^H NMR spectrum (Figure 2C and Figure S13). The chemical shifts are also consistent with the guest moving within the cavity.

**Figure 3:**
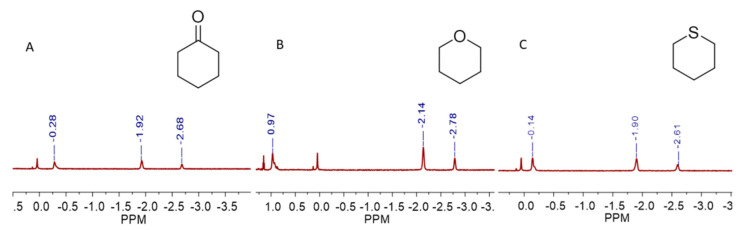
Partial ^1^H NMR spectra of cavitand **1** upon adding from left to right (**A**): cyclohexanone, (**B**): tetrahydropyran, (**C**): thiane.

Interestingly, we found that cavitand **1** shows higher binding affinity to hydrophilic cyclic molecules, compared to their hydrophobic counterparts of similar sizes and shapes, using competitive binding experiments. Cavitand **1** can selectively recognize hydrophilic cyclic molecules from their mixture with hydrophobic analogues. In the mixture of methylcyclohexane and cyclohexanecarboxylic acid, cavitand **1** will exclusively bind with cyclohexanecarboxylic acid. Similarly, in the mixture of cyclohexane and its functionalized derivatives, such as cyclohexanol and cyclohexylamine, only the functionalized derivatives can be bound by cavitand **1** (Figure 2 and Figures S17–S19). Why does cavitand **1** exhibit stronger binding affinity to guests with higher hydrophilicity? This seems contradictory to the usual experience that hydrophobicity is the main driving force that attracts the guest into the cavitand and the solvation of the hydrophilic groups by water. One might attribute this selectivity to the synergistic action of hydrogen bonding and the hydrophobic effect, which drives the hydrophilic molecules into cavitand **1**. According to Figure 1, the cavitand has two regions: On the upper rim, the amides provide abundant hydrogen bond donors and receptors for the guest retained in the cavitand, which we named hydrogen bonding region. The remaining framework of **1,** which comprises 4 aromatic walls and the resorcinarene core, provides an π electron-rich cavity we named the hydrophobic region. For nonpolar guests, such as cyclohexane and methylcyclohexane, the hydrophobic effect is the main driving force for the complexation. When molecules with both nonpolar parts and polar functions are encountered, cavitand **1** can provide a hydrophobic environment to accommodate the hydrophobic surface of the guests, and the amides on the upper rim will supply hydrogen bond donors and acceptors for the guests’ polar functional groups. Such explanations can be further supported by the binding of cyclohexanone or heterocyclic molecules such as tetrahydropyran and thiane in **1**, guests that have both hydrophobic parts and polar groups and exhibit fairly good water solubility. The ^1^H NMR spectrum indicates these guests are bound by cavitand **1** in the expected way, where the nonpolar parts are surrounded by the hydrophobic cavity and the polar groups are positioned to the open end and interact with the hydrogen bonding sites provided by cavitand’s upper rim (Figure 3 and Figures S20–S22). The furthest upfield signals (at about −2.7 ppm) indicate that a -CH_2_- group is fixed at the bottom of the cavity since it experiences the maximum upfield shift (Δδ = −4 ppm). 

The hydrophilic groups of the intended guests are, of course, well-solvated by water—so why does the guest prefer the amides of the host? The answer may lie in the nature of solvation. In bulk solution, solvent water molecules must surround the functional groups of solutes in proper orientations. This limits their translational motions, at some considerable entropic cost. The amides of the cavitand rim are already fixed in place; they are not free to “get away” since the price of their free translation has been paid for during the synthesis. The amides are a fixed solvent cage ready for polar functional groups on guests. Given that secondary amides and water have comparable hydrogen bond donating and accepting abilities, the preorganization of the cavitand’s rim gives it an entropic advantage for solvating polar functions of the guests.

**Figure 4:**
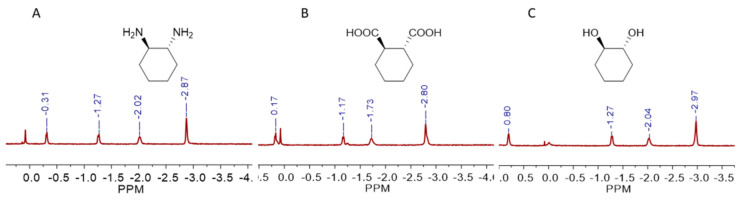
Partial ^1^H NMR spectra of cavitand **1** upon adding from left to right (**A**): *trans*-1,2-diaminocyclohexane, (**B**): *trans*-1,2-cyclohexanedicarboxylic acid, (**C**): *trans*-1,2-cyclohexanediol.

More hydrophilic molecules such as disubstituted cyclohexane derivatives can also be recognized by cavitand **1**. We examined the binding of *trans*-1,2-diaminocyclohexane, *trans*-1,2-cyclohexanedicarboxylic acid and *trans*-1,2-cyclohexanediol, and the ^1^H NMR spectra showed all these hydrophilic molecules can be bound in a 1:1 stoichiometry (Figure 4 and Figures S23–S25). Like the monosubstituted cyclohexane derivatives, these disubstituted molecules also buried their nonpolar part deeply in the hydrophobic cavity and placed the polar groups on the open end of **1**. We also tested the binding of adamantane and a series of its monosubstituted derivatives, and **1** shows excellent binding ability to the adamantane derivatives. For adamantane itself, only weak signals of host–guest complex were observed; even the sample was sonicated for a long time (Figure S26).

**Figure 5:**
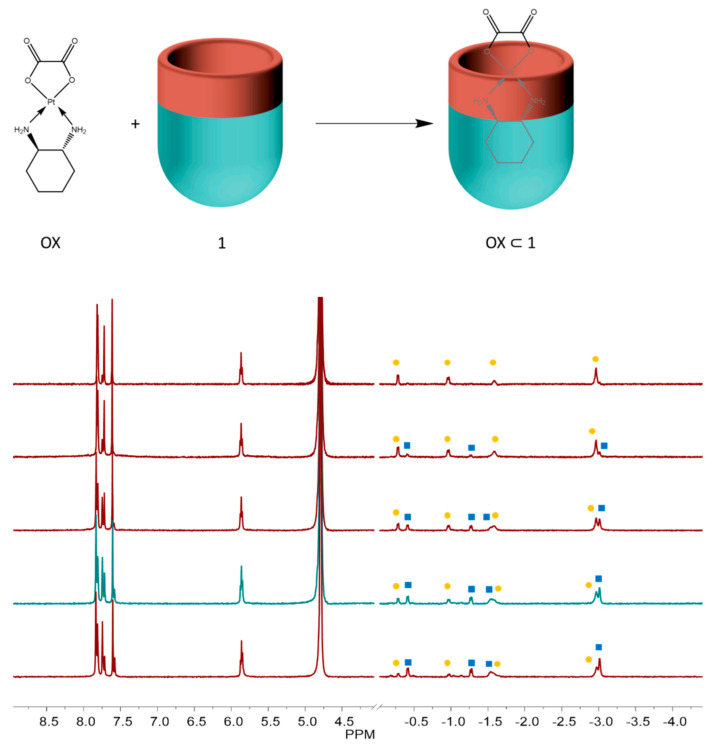
**Top**: Formation of host–guest complex by mixing an equimolar amount of oxaliplatin and cavitand **1** in water. **Bottom**: Partial ^1^H NMR spectra of the host–guest complex formed between equimolar oxaliplatin and cavitand **1** in D_2_O, as a function of time: for from bottom to top 1 h, 12 h, 24 h, 4 days and 7 days. The guest exhibits two group of peaks labeled with dots and squares, respectively.

Finally, a number of supramolecular hosts have been introduced as drug carriers to increase a drug’s stability, water solubility, biocompatibility, tumor selectivity and to decrease the drugs’ toxicity [28,29,30]. Oxaliplatin is a third-generation platinum anticancer drug with advantages compared with cisplatin and carboplatin, including a broad range of application in tumors, low toxicity and little or no cross-resistance to cisplatin [31,32,33]. But oxaliplatin has some drawbacks such as fast degradation in the blood stream. Since the structure of oxaliplatin contains a diaminocyclohexane component that fits well to the cavity of **1**, we checked its binding behavior. The large upfield shifted signals of bound guest in ^1^H NMR spectrum indicates the diaminocyclohexyl ligand of oxaliplatin is embedded deeply into the bottom of **1** with the oxalate function near the open end of the cavitand (Figure 5). Interestingly, two groups of signal peaks of the cyclohexyl of oxaliplatin bound in cavitand **1** were observed in the ^1^H NMR spectrum; with time, one group of peaks (labeled with squares) disappeared and the other group of peaks (labeled with dots) increased correspondingly. However, the nature of the transformation is not clear and needs further study.

## 3. Materials and Methods

### 3.1. Chemicals

All analytical grade solvents and reagents purchased from commercial sources were used without further purification. Acetyl chloride, sodium iodide and trimethylamine solution in ethanol were purchased from J&K Chemical Company Ltd., Shanghai, China, Meryer (Shanghai) Chemical Technology Co., Ltd. (Shanghai, China) and Shanghai Macklin Biochemical Co., Ltd. (Shanghai, China) respectively.

### 3.2. Synthesis of Cavitand 2

Octa-amino cavitand (Cav-8NH_3_Cl, 1.36 g, 1 mmol) was suspended in a round bottom flask containing 20 mL of dichloromethane, 8 equivalents of triethylamine was added by using a syringe with vigorous stirring and the mixture was further stirred for 10 min at rt.; 16 equivalents of acetyl chloride was added to it dropwise with constant stirring. Upon adding acetyl chloride, the solid will be dissolved and the mixture will become a clear pale yellow solution. After stirred at rt. for 48 h, the solvent was removed by using a rotary evaporator under reduced pressure; the raw product was purified by silica gel column chromatography using dichloromethane/methanol (*v/v* = 30:1) as eluent to afford cavitand 2. Yield: 1.04 g/67%. ^1^H NMR (600 MHz, Chloroform-d) δ 10.00 (s, 4H), 9.22 (s, 4H), 7.78 (s, 4H), 7.32 (s, 4H), 7.24 (s, 8H), 5.82 (t, *J* = 8.2 Hz, 4H), 3.70 (t, *J* = 6.2 Hz, 8H), 2.44–2.48 (m, 8H), 2.26 (s, 12H), 2.13 (s, 12H), 1.85–1.90 (m, 8H). ^13^C NMR (600 MHz, Chloroform-*d*/DMSO-*d*_6_ (9:1)) δ 169.75, 154.68, 149.51, 135.17, 127.91, 123.96, 120.90, 116.23, 44.98, 32.79, 31.08, 29.29, 24.48. HR-MS (ESI): Calcd. for chemical formula C_80_H_76_Cl_4_N_8_O_16_:1544.4133, found: 1545.4236 [M+H]^+^.

### 3.3. Synthesis of Cavitand 3

Cavitand 2 (154.7 mg, 0.1 mmol) and sodium iodide (200 mg, 2 mmol) were dissolved in 10 mL of acetone in a round bottom flask; the mixture was equipped with a magnetic stir bar and refluxed for 3 days. The solvent was removed by using a rotary evaporator and 20 mL of dichloromethane was added to the residue; the mixture was briefly sonicated and then filtered through a glass filter. The filtrate was collected and the solvent was removed by rotary evaporation to afford the cavitand 3 as a white solid. Yield: 172 mg/90%. ^1^H NMR (600 MHz, Chloroform-*d*) δ 10.03 (s, 4H, N–H), 9.23 (s, 4H, N–H), 7.80 (s, 4H), 7.42 (s, 4H), 7.28 (s, 8H), 5.86 (t, *J* = 8.2 Hz, 4H), 3.39 (t, *J* = 6.2 Hz, 8H), 2.47–2.55 (m, 8H), 2.28 (s, 12H), 2.16 (s, 12H), 1.88–1.93 (m, 8H). ^1^H NMR (600 MHz, Chloroform-*d*/DMSO-*d*_6_ (9:1)) δ 9.59 (s, 8H, N–H), 7.52 (s, 8H), 7.45 (s, 8H), 5.83 (t, *J* = 7.9 Hz, 4H), 3.37 (t, *J* = 6.7 Hz, 8H), 2.56–2.46 (m, 8H), 2.21 (s, 24H), 1.86–1.96 (m, 8H). ^13^C NMR (600MHz, CDCl_3_/DMSO-*d*_6_ (9:1)) δ 169.76, 154.70, 149.50, 135.06, 127.92, 123.71, 120.89, 116.34, 33.14, 32.49, 31.60, 30.90, 24.49. HR-MS (ESI): Calcd. for chemical formula C_80_H_76_I_4_N_8_O_16_: 1912.1558, found: 1913.1638 [M+H]^+^.

### 3.4. Synthesis of Cavitand 1

Cavitand 3 (153 mg, 0.08 mmol) was suspended in 10 mL of trimethylamine solution (30 wt. %) in ethanol in a round bottom flask; the mixture was equipped with a magnetic stir bar and stirred at 50 °C for 12 h. Then the mixture was cooled to room temperature and the precipitate was collected through filtration and washed with 40 mL of ethanol for two times, and then dried under high vacuum. Yield: 154 mg/90%. ^1^H NMR (600 MHz, DMSO-*d*_6_) δ 9.21–9.60 (m, 8H, N–H), 7.41–8.00 (m, 16H, Ar–H), 5.67 (s, 4H), 3.55 (s, 8H), 3.07 (s, 36H), 2.64 (s, 8H), 2.10 (s, 24H), 1.67 (s, 8H). ^13^C NMR (600 MHz, DMSO) δ 168.90, 154.19, 147.66, 135.38, 128.33, 125.19, 120.19, 116.35, 65.64, 52.43, 33.51, 29.07, 23.90, 20.80. HR-MS (ESI): Calcd. for chemical formula C_92_H_112_N_12_O_16_ [M-4I^−^]/4 410.2080, found: 410.2074[M-4I]^4+^/4.

### 3.5. General Procedure for the Binding Analyses

A 1 mM, 0.5 mL solution of cavitand 1 in D_2_O was taken in NMR tube and excess pure guest (~0.25 µL) or 5 µL of guest stock solution in D_2_O (100 mM) was added to the tube; it was shaken well to mix the guest in water. The sample was sonicated briefly and analyzed by ^1^H NMR spectroscopy.

## 4. Conclusions

In summary, a cationic water-soluble deep cavitand was prepared and characterized, which bears octa amides on the upper rim and tera trimethylammonium groups on the feet. This kind of water-soluble cavitand can selectively recognize hydrophilic six-membered cyclic molecules in water through the synergistic action of hydrogen bond and hydrophobic effect. Moreover, the cavitand also exhibits excellent binding ability for a specific anticancer drug oxaliplatin and may act as a potential carrier for such an anticancer drug.

## Data Availability

Data are contained within the article or Supplementary Material.

## Supplementary Materials

The following are available online. Figures S1–S8: the ^1^H NMR and ^13^C NMR spectra of the cavitands, Figures S9–S11: the Mass spectra of the cavitands, Figures S12–S17: binding properties of cavitand 1 to different guests.

## Sample Availability

Samples of the compounds are not available from the authors.
