# Supplementary material for: Recognition of Hydrophilic Cyclic Compounds by a Water-Soluble Cavitand
Source: Molecules. 2021 Mar 30;26(7):1922. doi: 10.3390/molecules26071922 (PMC8037811; doi:10.3390/molecules26071922)
Supplement: Supplementary file 1 [file molecules-26-01922-s001.pdf]

Supplementary Materials

Recognition of Hydrophilic Cyclic Compounds by a Water-soluble Cavitand

Yun-huiWan[1], Yu-jie Zhu[1], Julius Rebek, Jr.[2], Yang Yu*[1]

[1] Center for Supramolecular Chemistry & Catalysis and Department of Chemistry, College of Science, Shanghai University, 99 Shang-Da Road, Shanghai 200444, China
[2] Skaggs Institute for Chemical Biology and Department of Chemistry, The Scripps Research Institute, 10550 North Torrey Pines Road, La Jolla, CA 92037, USA

* Correspondence: *yangyu2017@shu.edu.cn*

Content

^{1}H NMR, ^{13}C NMR spectra of the cavitands……………………………………………………1

Mass (HR) spectra of cavitands………………………………………………………………..............5

^{1}H NMR spectra of the host-guest complex formed between cavitand **1** with different guests……………………………………………………………………………………………….................8

^{1}H NMR, ^{13}C NMR spectra of the cavitands

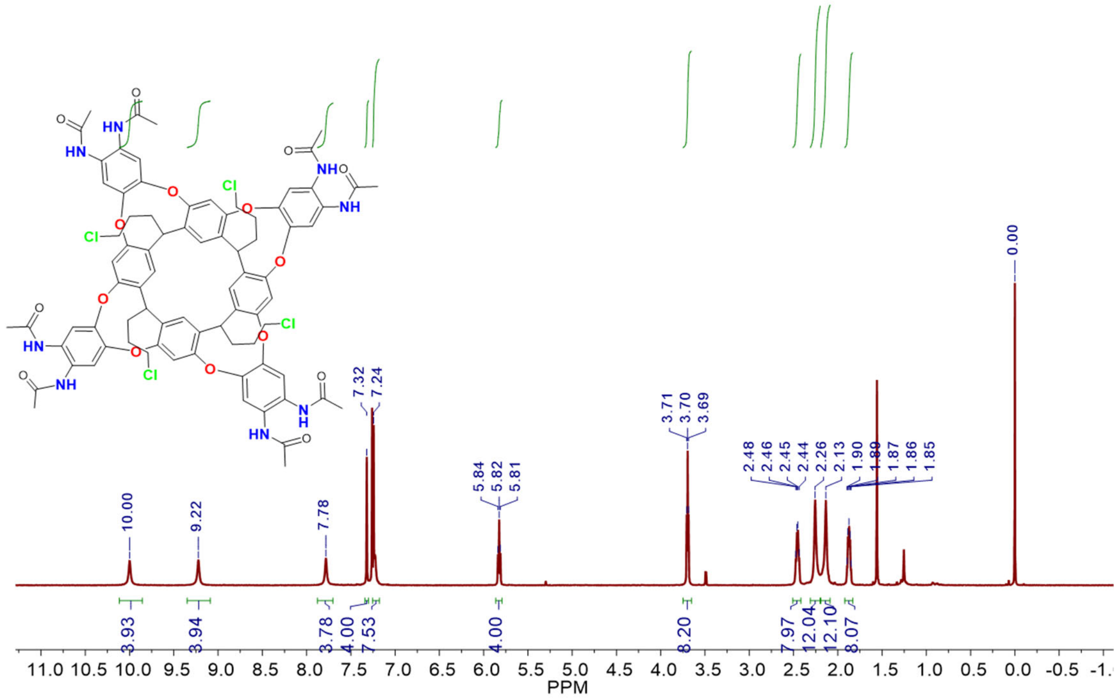

Fig. S1 ^{1}H NMR spectrum of cavitand **3** in chloroform-*d* at rt

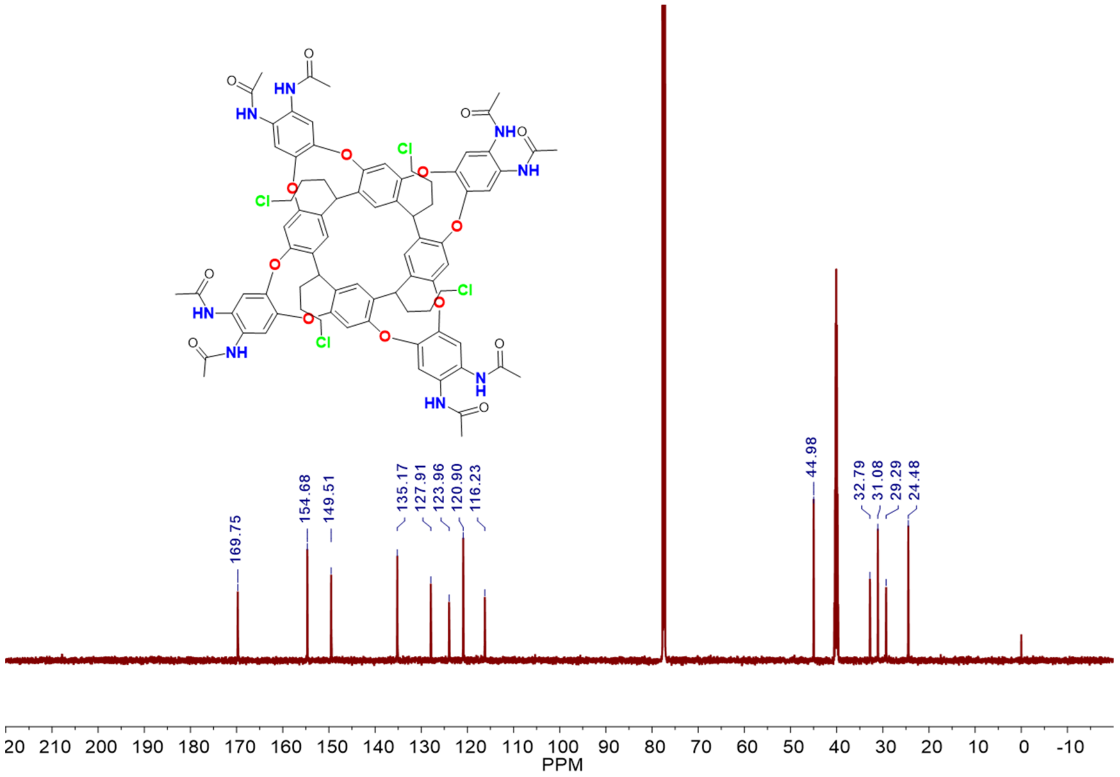

Fig. S2 ^{13}C NMR spectrum of cavitand **3** in chloroform-*d*/DMSO-d_6 (vol/vol = 9 :1) at rt

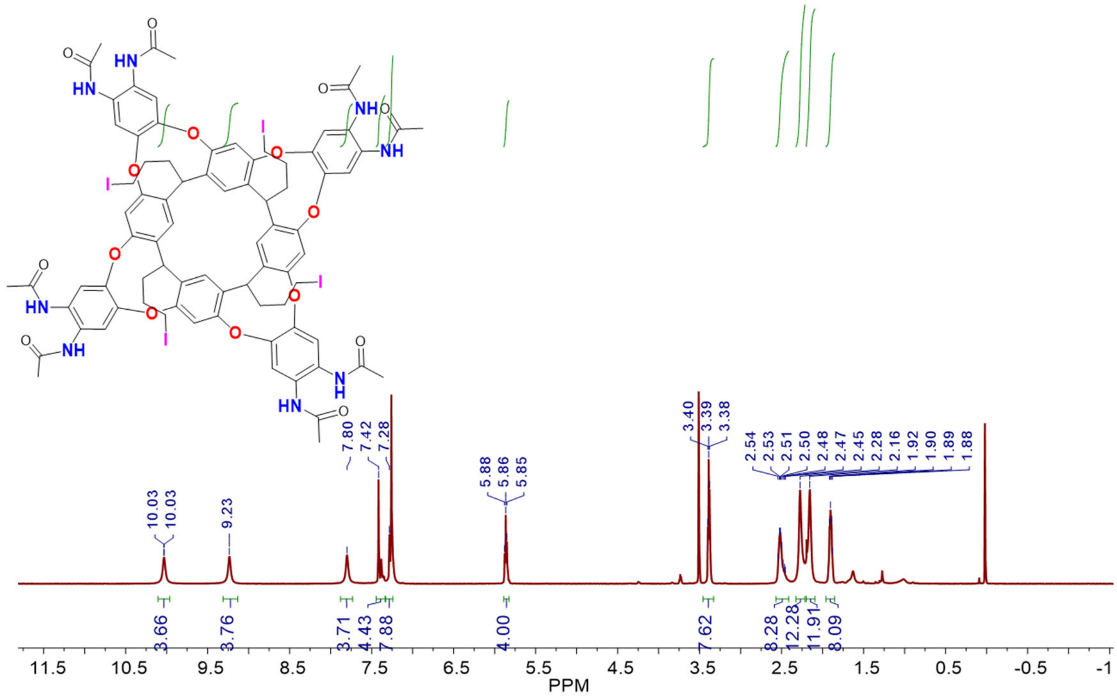

Fig. S3 ^{1}H NMR spectrum of cavitand **2** in chloroform-*d* at rt

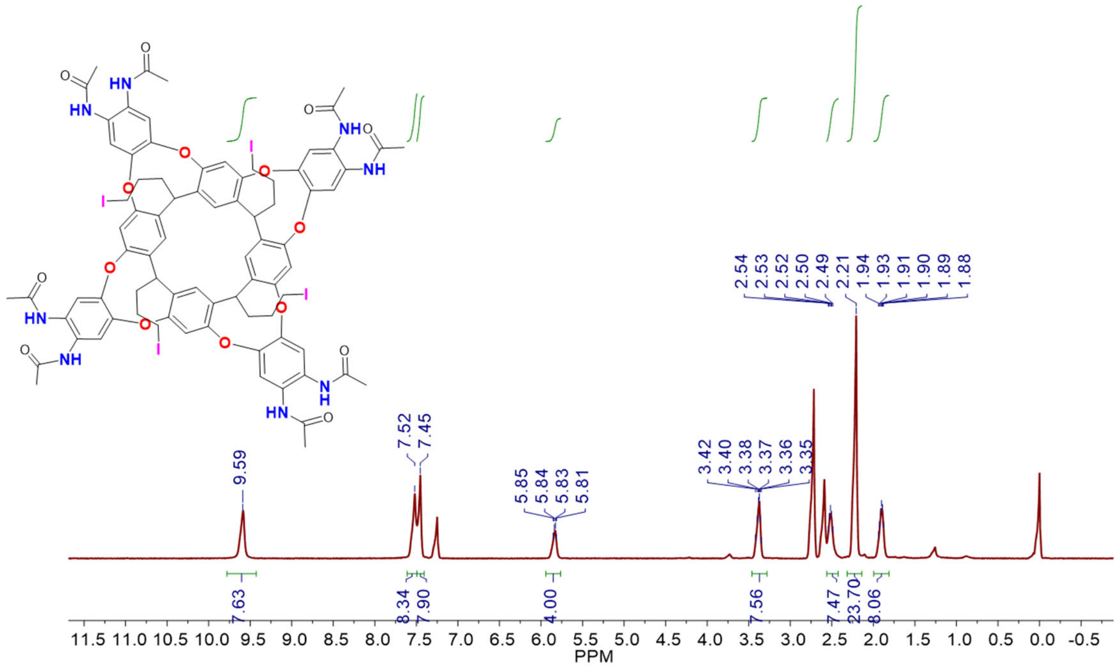

Fig. S4 ^{1}H NMR spectrum of cavitand **2** in chloroform-*d*/DMSO-*d*$_6$ (vol/vol = 9 :1) at rt

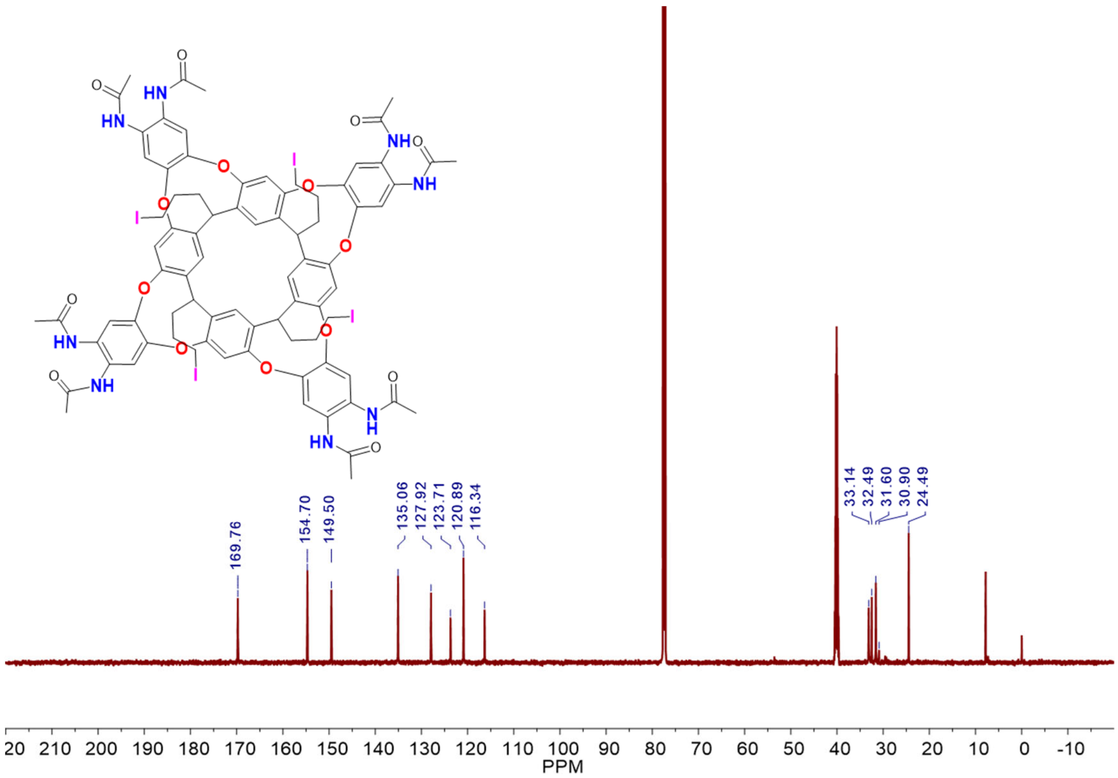

Fig. S5 ^{13}C NMR spectrum of cavitand **2** in chloroform-*d*/DMSO-*d*6 (vol/vol = 9 :1) at rt

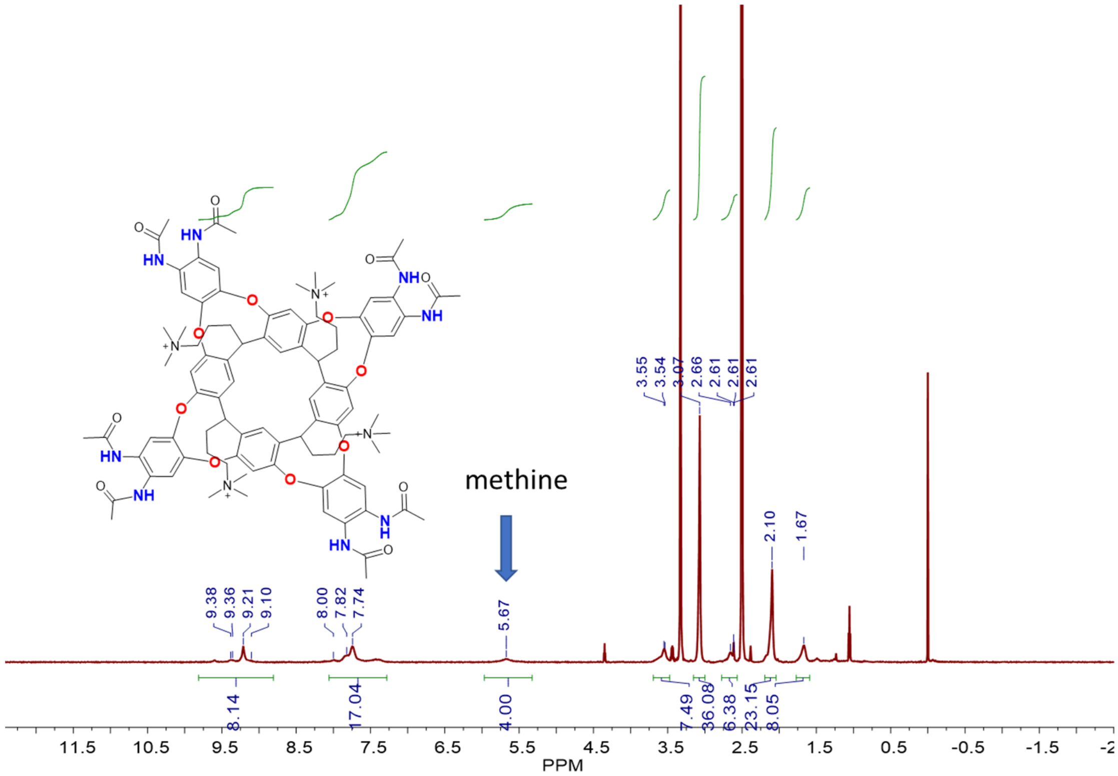

Fig. S6 ^{1}H NMR spectrum of cavitand **1** in DMSO-*d*6 at rt, the cavitand display vase form exclusively

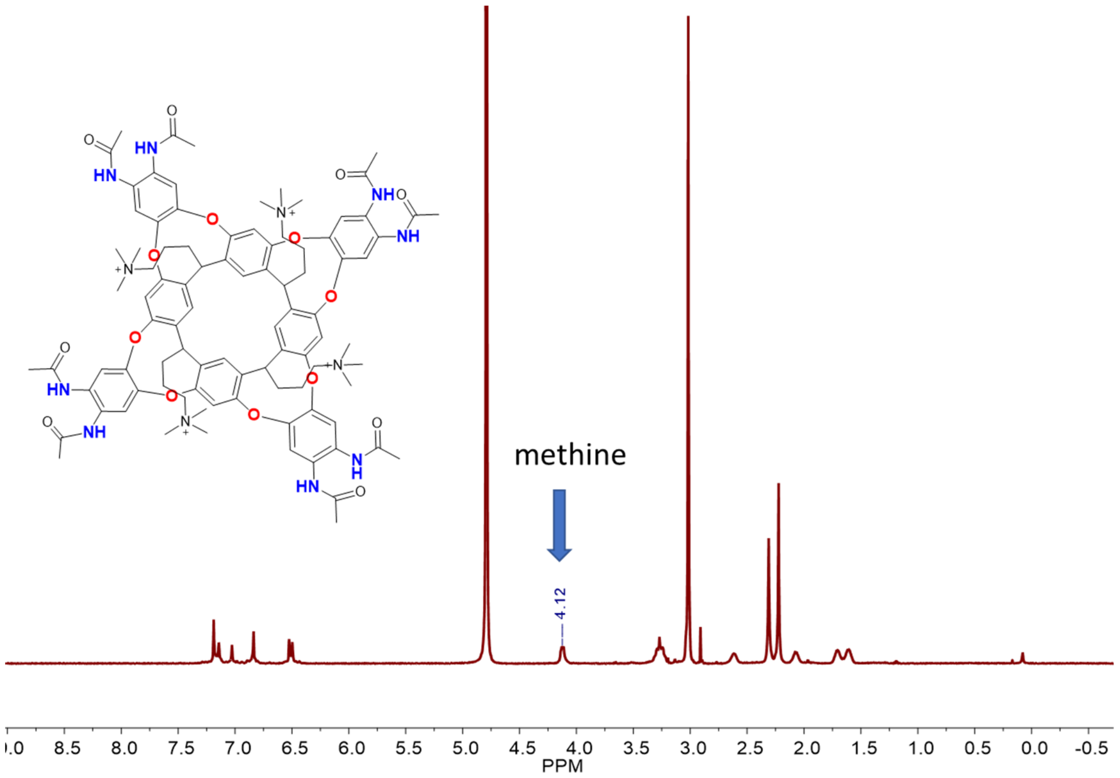

Fig. S7 ^{1}H NMR spectrum of cavitand **1** in D_2O at rt, the cavitand display kite form exclusively

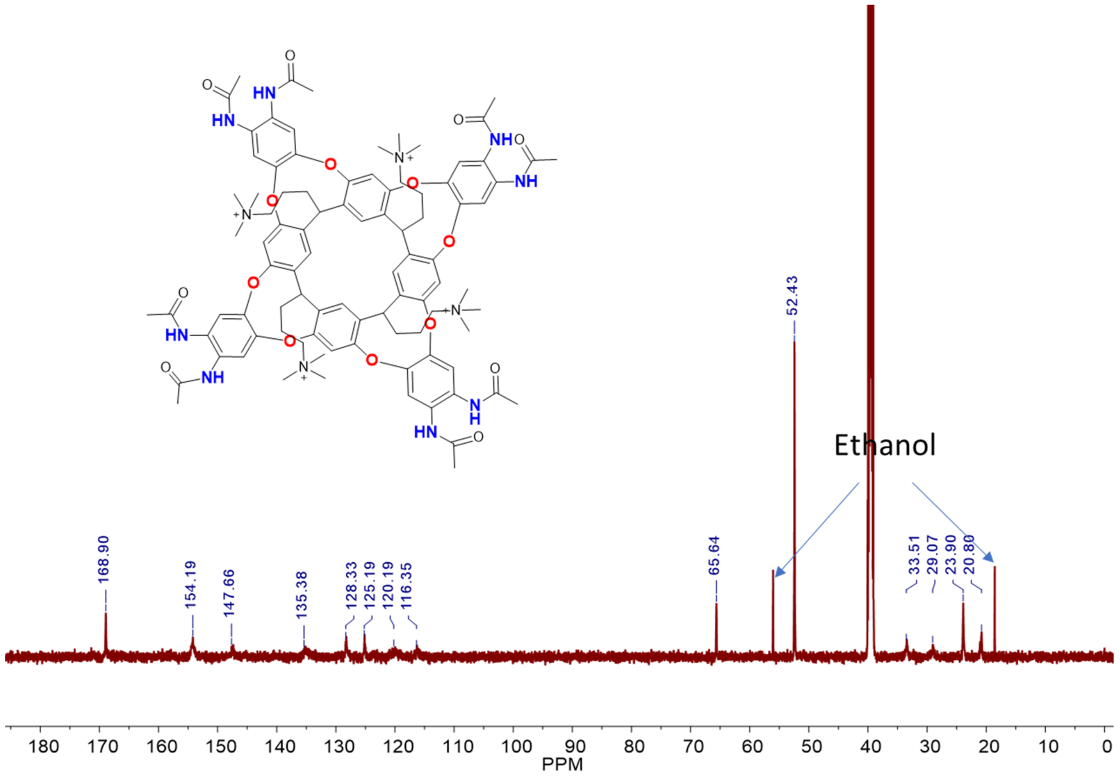

Fig. S8 ^{13}C NMR spectrum of cavitand **1** in DMSO-d_6 at rt

Mass (HR) spectra of cavitands

Display Report

Analysis Info

Analysis Name	D:\Data\2020MS\yy\1123\4D-8-AMIDE-CAVITAND_BE3_01_3071.d	Acquisition Date	11/24/2020 11:41:48 AM
Method	apci_wide_500-3000_pos-6min.m	Operator	gftang
Sample Name	4D-8-AMIDE-CAVITAND	Instrument / Ser#	micrOTOF 10257
Comment			

Acquisition Parameter

Source Type	APCI	Ion Polarity	Positive	Set Nebulizer	2.0 Bar
Focus	Not active			Set Dry Heater	200 °C
Scan Begin	300 m/z	Set Capillary	4000 V	Set Dry Gas	4.0 l/min
Scan End	2500 m/z	Set End Plate Offset	-500 V	Set Divert Valve	Waste

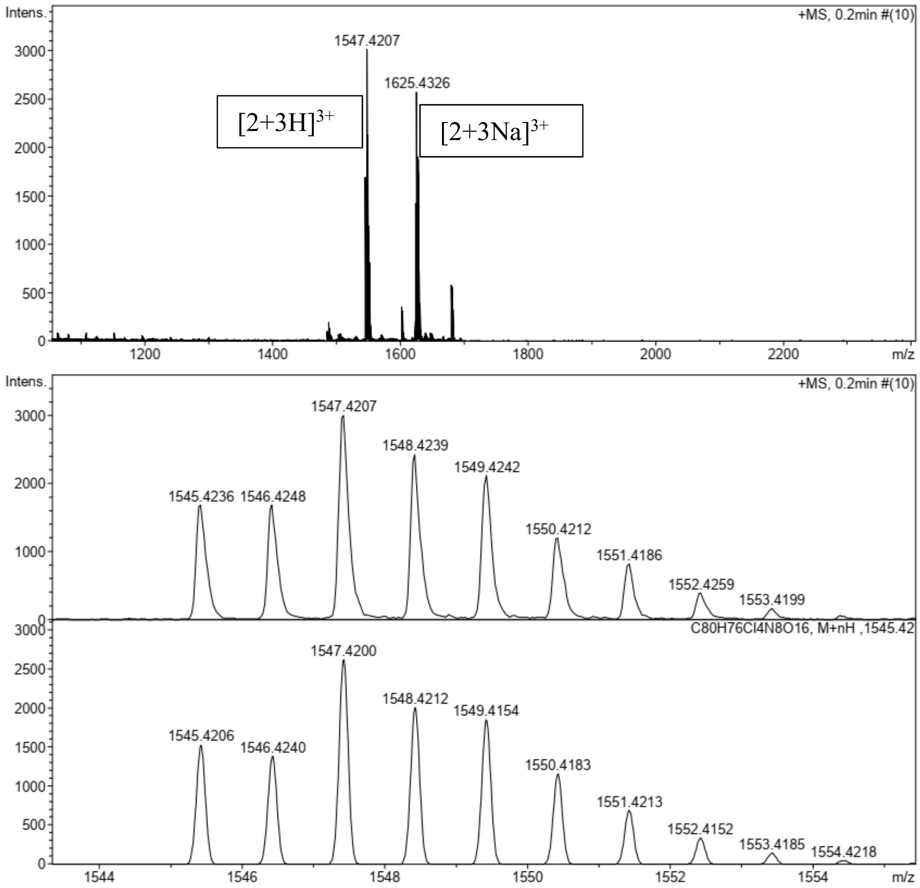

Bruker Compass DataAnalysis 4.0 printed: 11/24/2020 11:50:52 AM Page 1 of 1

Fig. S9 Mass spectrum of cavitand **2**

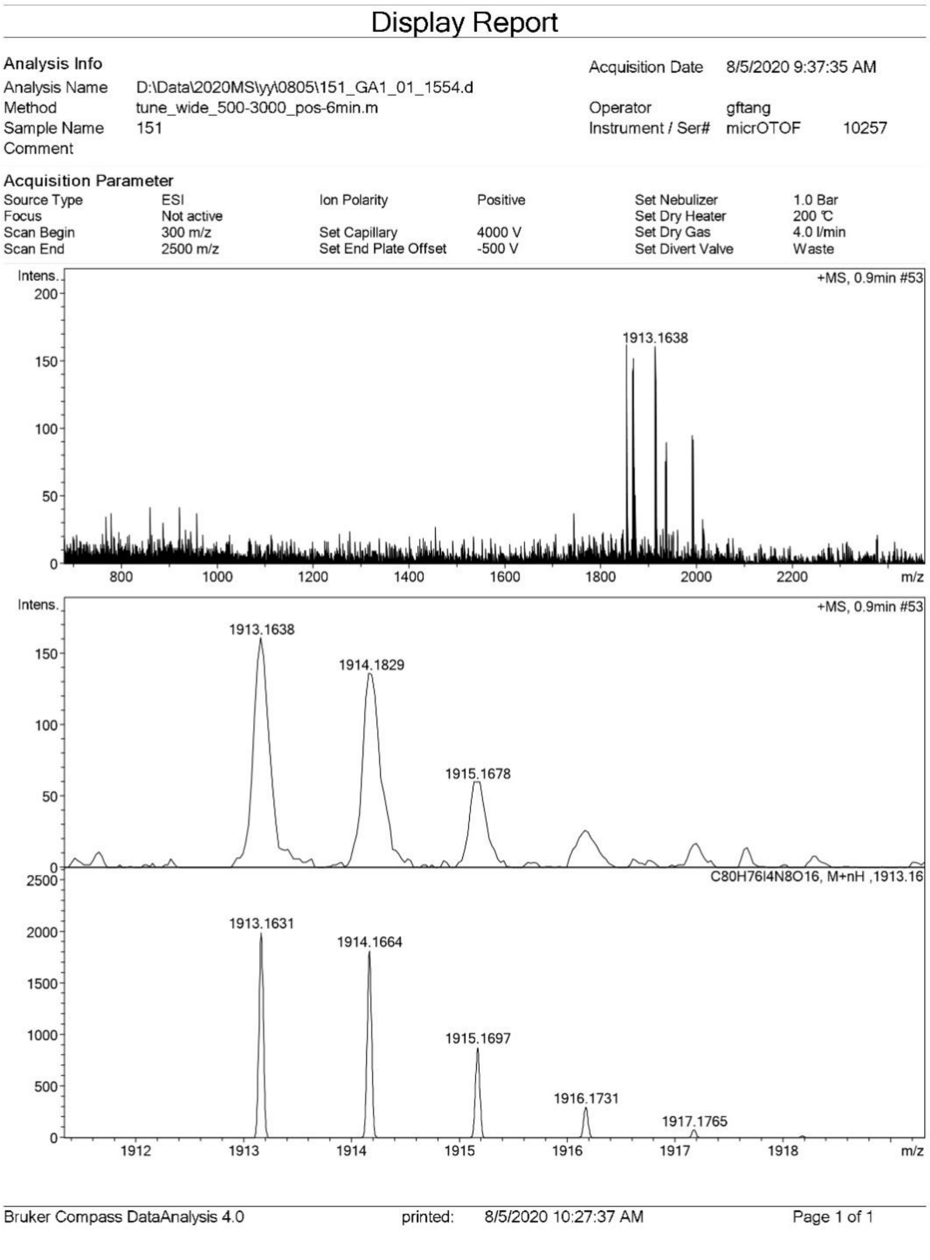

Fig. S10 Mass spectrum of cavitand **3**

Display Report

Analysis Info

Analysis Name	D:\Data\2020MS\yy\0805\156_GA2_01_1552.d	Acquisition Date	8/5/2020 9:23:29 AM
Method	tune_wide_500-3000_pos-6min.m	Operator	gftang
Sample Name	156	Instrument / Ser#	micrOTOF 10257
Comment			

Acquisition Parameter

Source Type	ESI	Ion Polarity	Positive	Set Nebulizer	1.0 Bar
Focus	Not active			Set Dry Heater	200 ℃
Scan Begin	300 m/z	Set Capillary	4000 V	Set Dry Gas	4.0 l/min
Scan End	2500 m/z	Set End Plate Offset	-500 V	Set Divert Valve	Waste

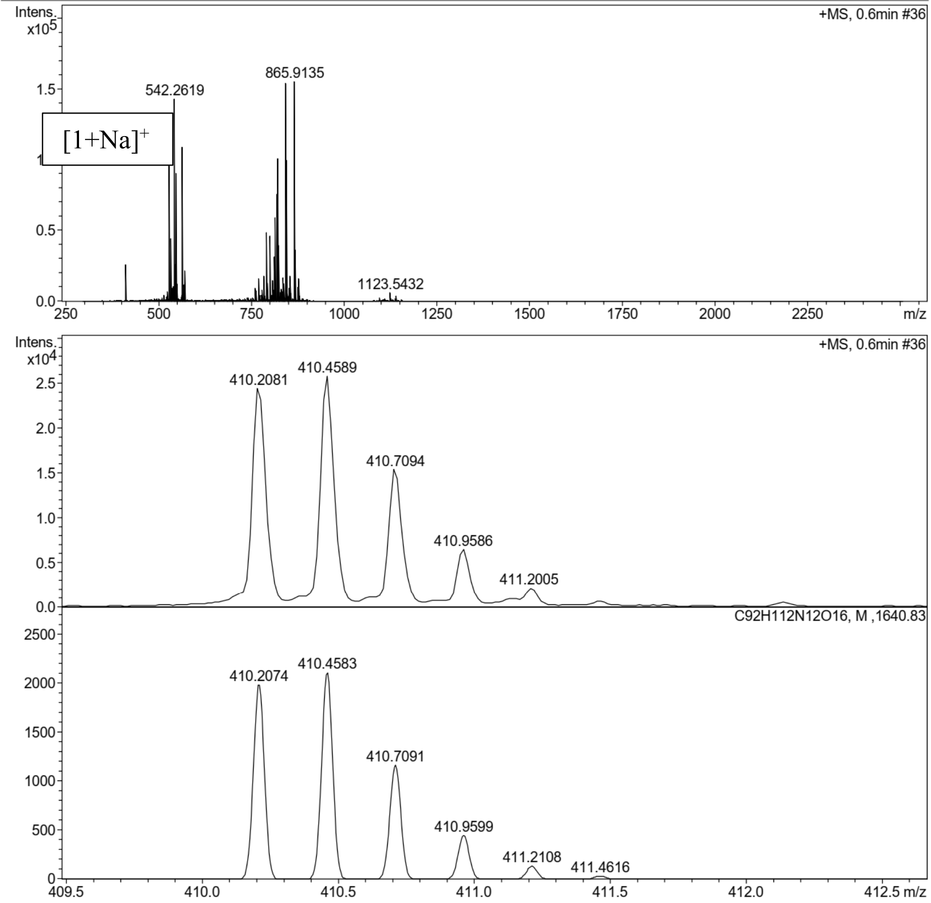

Bruker Compass DataAnalysis 4.0 printed: 8/5/2020 9:30:16 AM Page 1 of 1

Fig. S11 Mass spectrum of cavitand **1**

^{1}H NMR spectra of the host-guest complex formed between cavitand 1 with different guests

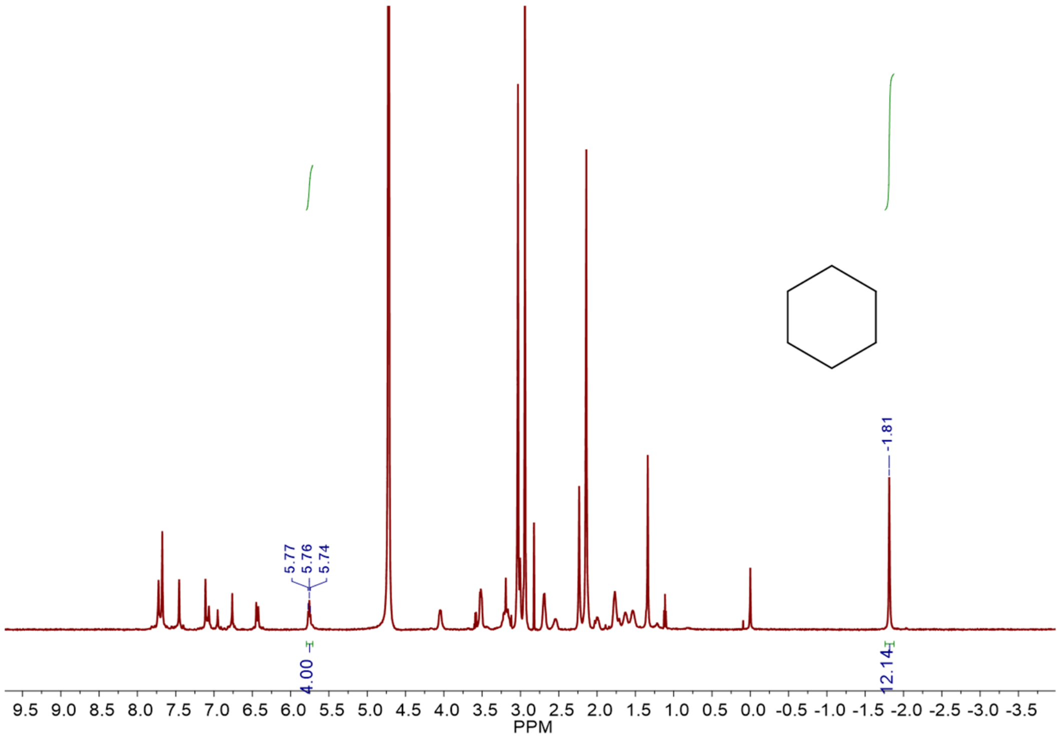

Fig. S12 ^{1}H NMR spectrum of the complex formed between cavitand **1** and excess of cyclohexane in D_2O

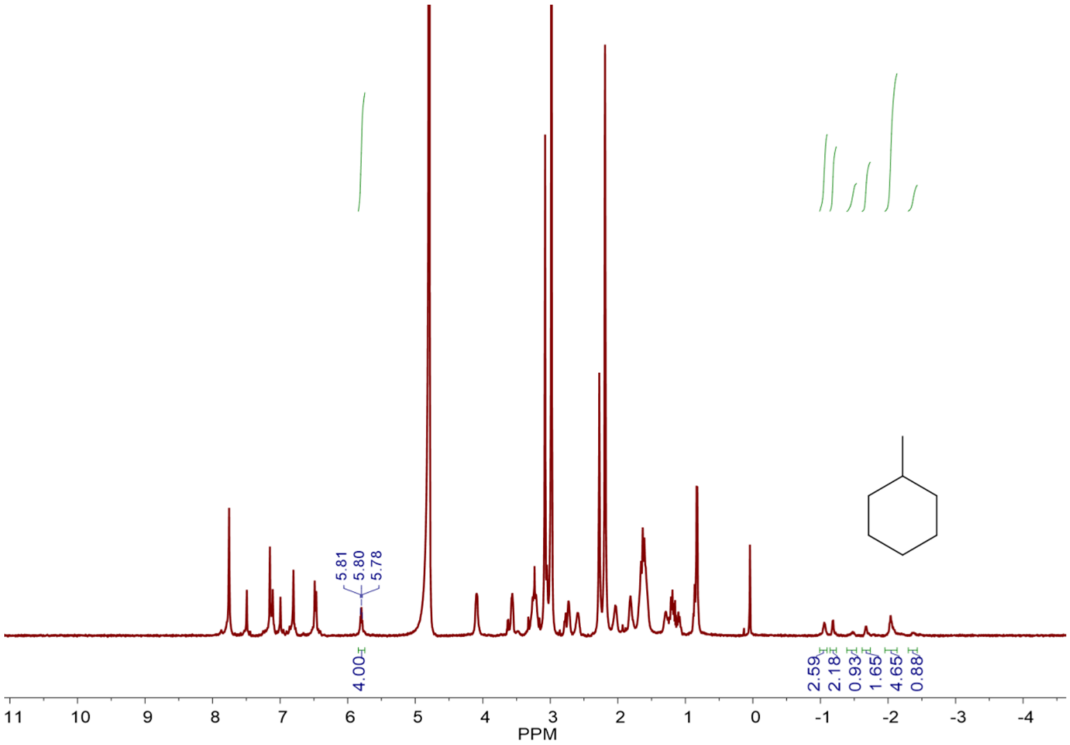

Fig. S13 ^{1}H NMR spectrum of the complex formed between cavitand **1** and excess of methylcyclohexane

in D_2O

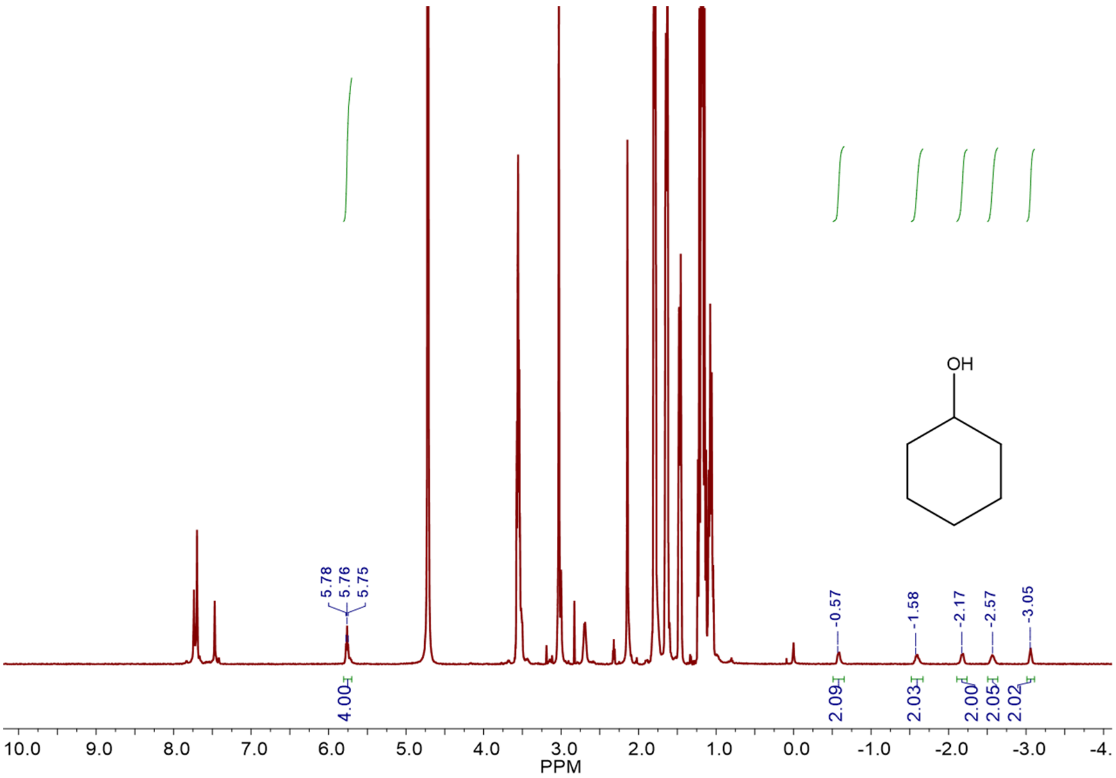

Fig. S14 ^{1}H NMR spectrum of the complex formed between cavitand **1** and excess of cyclohexanol in D_2O

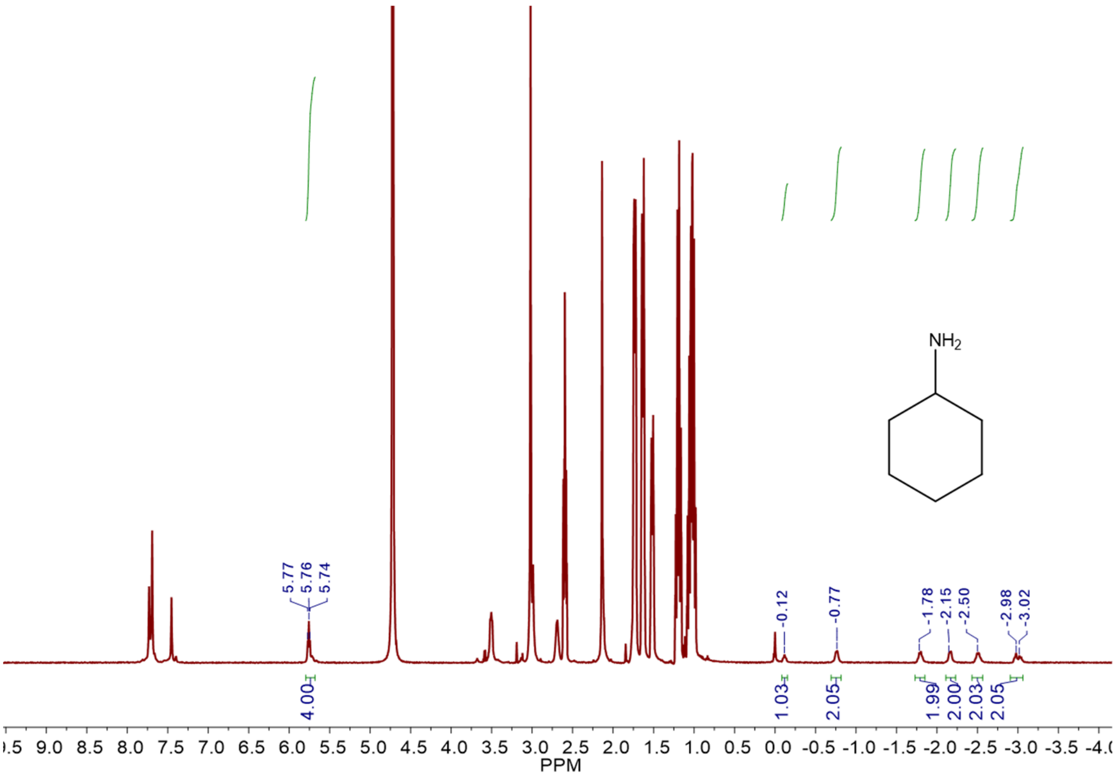

Fig. S15 ^{1}H NMR spectrum of the complex formed between cavitand **1** and excess of cyclohexylamine in

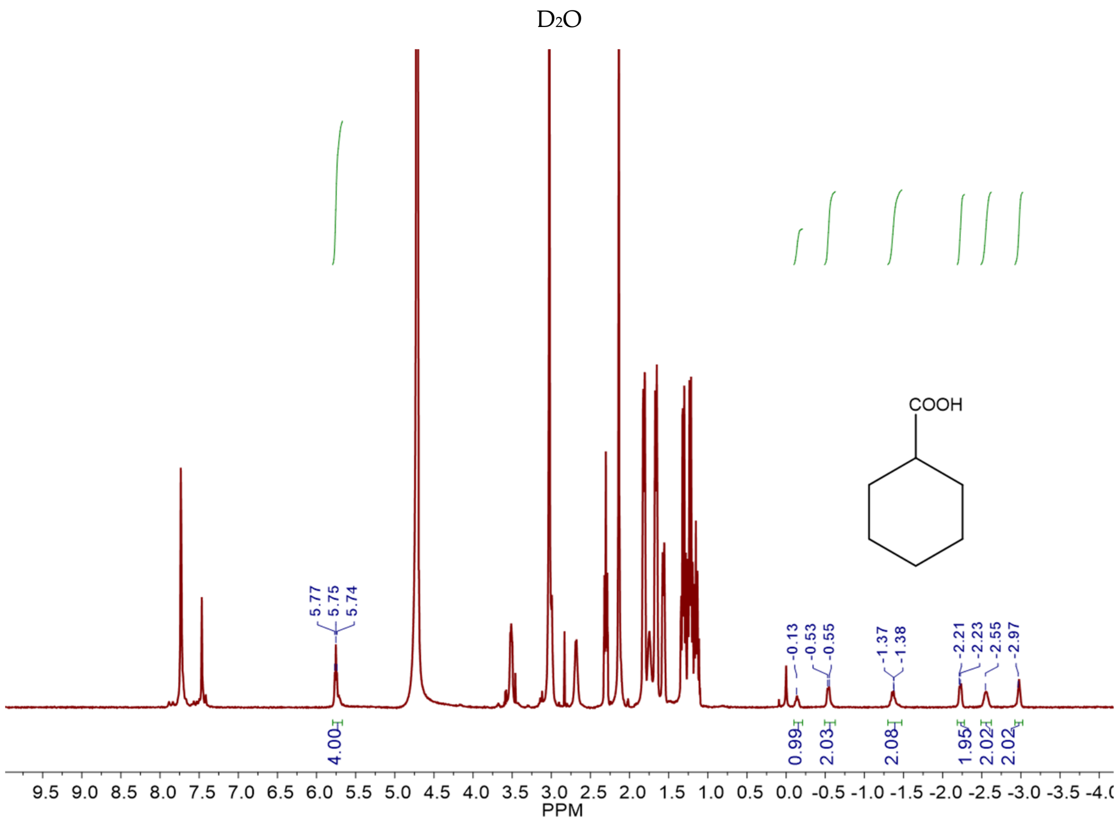

Fig. S16 ^{1}H NMR spectrum of the complex formed between cavitand **1** and excess of cyclohexanecarboxylic acid in D_2O

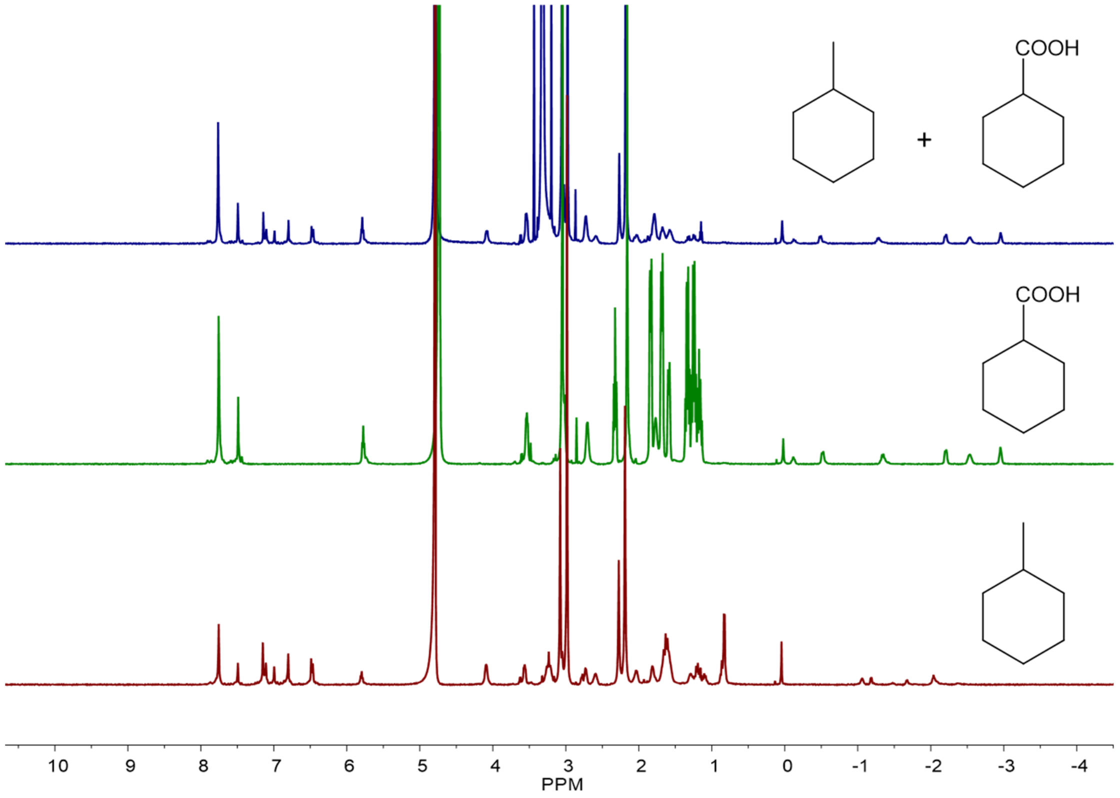

Fig. S17 ^{1}H NMR spectra of the complexes formed between cavitand **1**, 1mmol + from bottom to top, excess of methylcyclohexane, cyclohexanecarboxylic acid, and equimolar methylcyclohexane + cyclohexanecarboxylic acid (1:1) mixture.

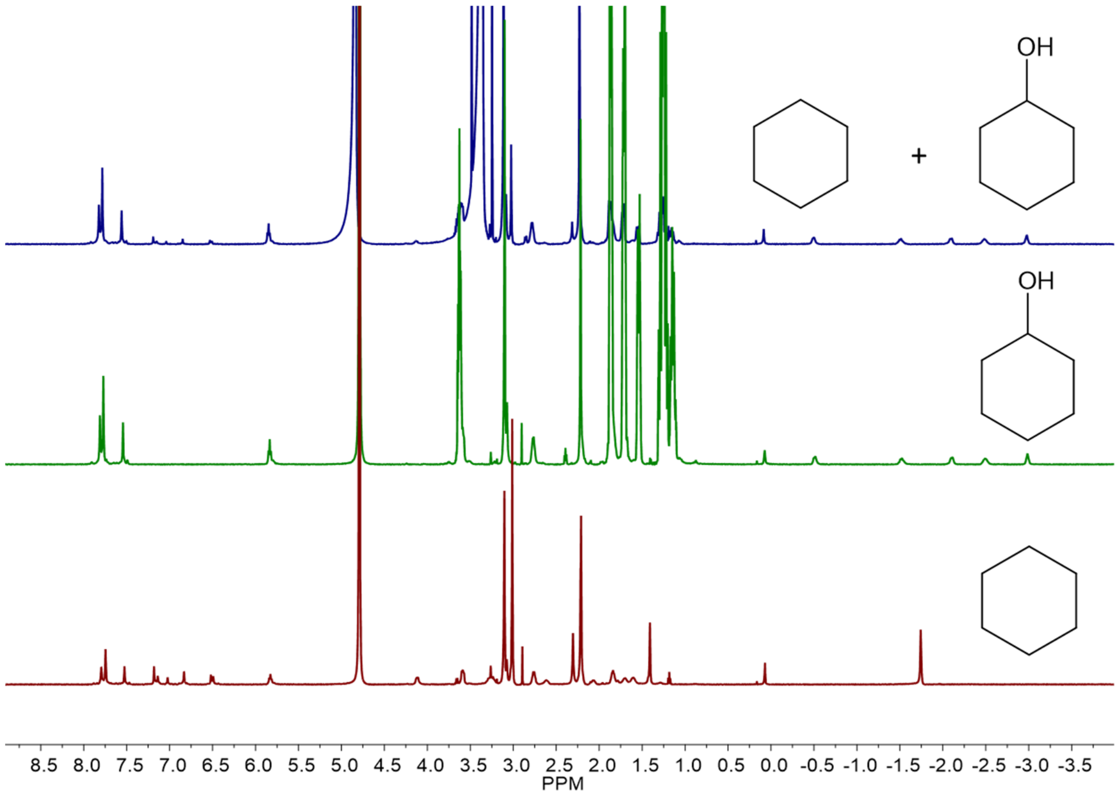

Fig. S18 ^{1}H NMR spectra of the complexes formed between cavitand **1**, 1mmol + from bottom to top, excess of cyclohexane, cyclohexanol, and equimolar cyclohexane + cyclohexanol (1:1) mixture.

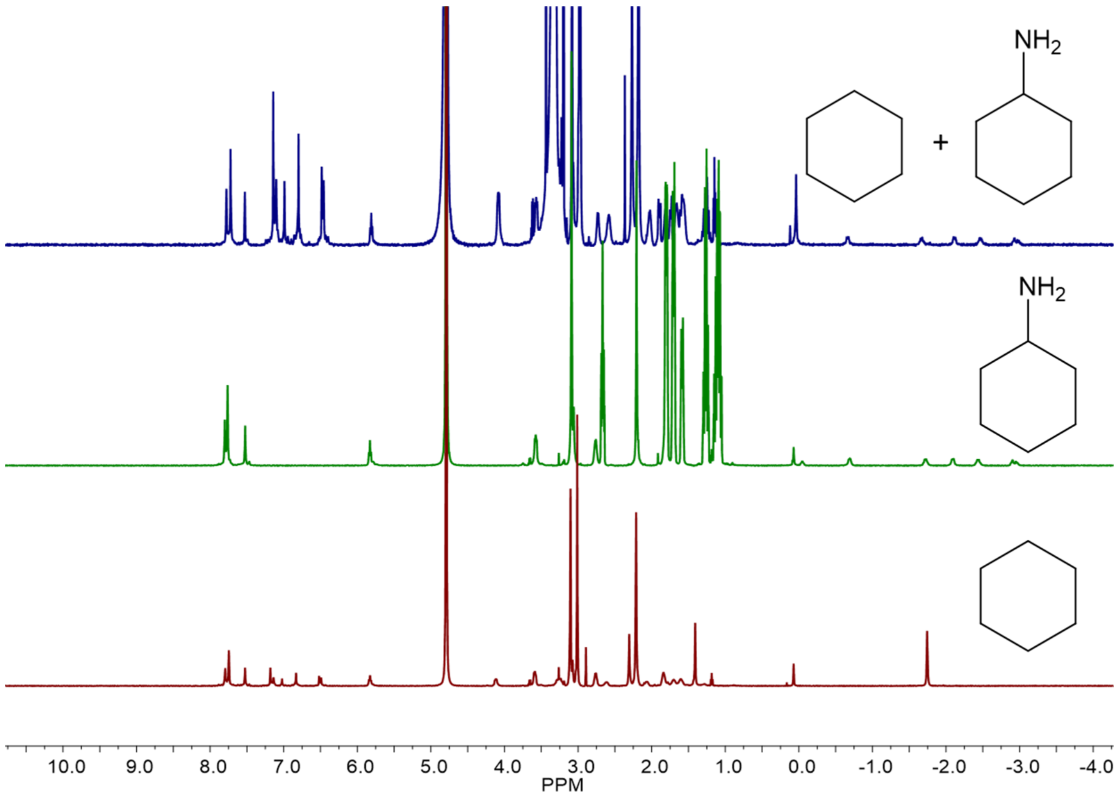

Fig. S19 ^{1}H NMR spectra of the complexes formed between cavitand **1**, 1mmol + from bottom to top, excess of cyclohexane, cyclohexylamine, and equimolar cyclohexane + cyclohexylamine (1:1) mixture

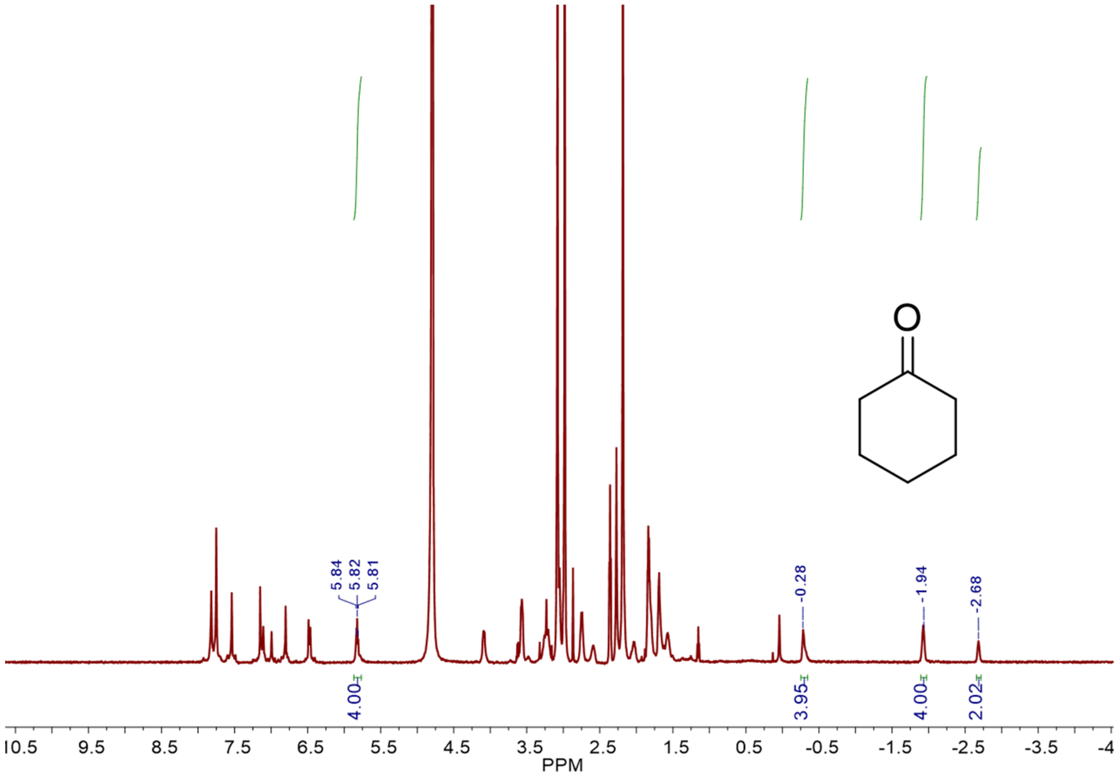

Fig. S20 ^{1}H NMR spectrum of the complex formed between cavitand **1** and excess of cyclohexanone in D_2O

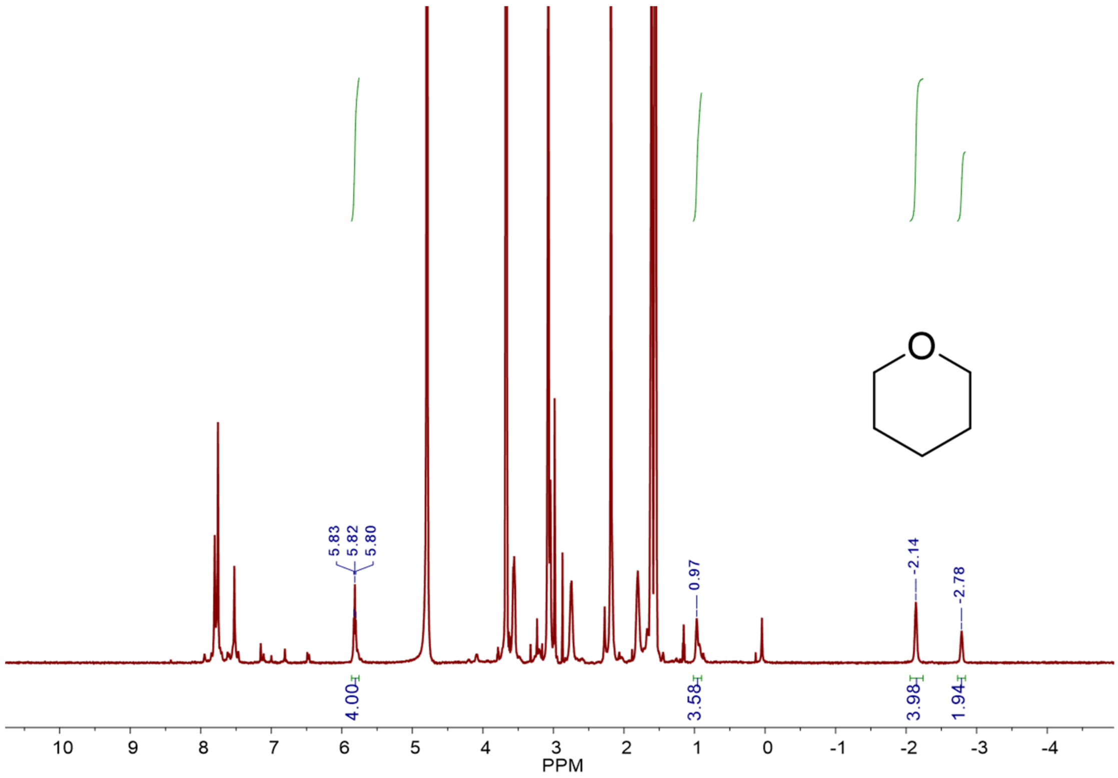

Fig. S21 ^{1}H NMR spectrum of the complex formed between cavitand **1** and excess of tetrahydropyran in D_2O

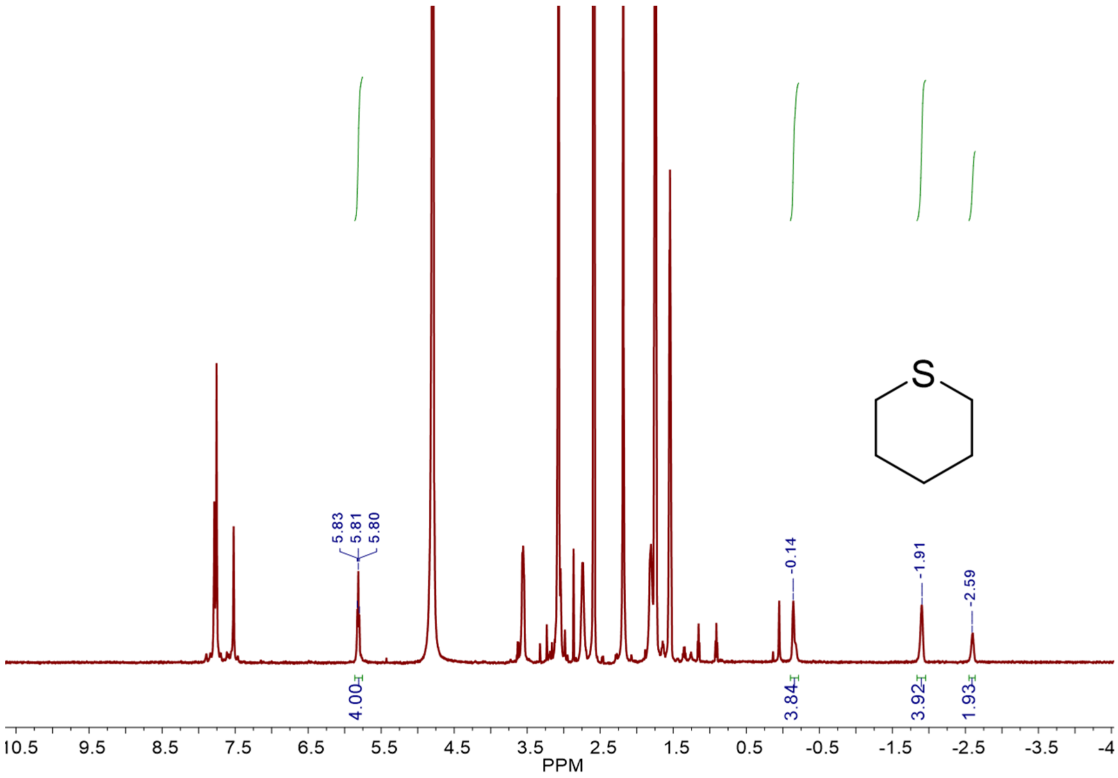

Fig. S22 ^{1}H NMR spectrum of the complex formed between cavitand **1** and excess of thiane in D_2O

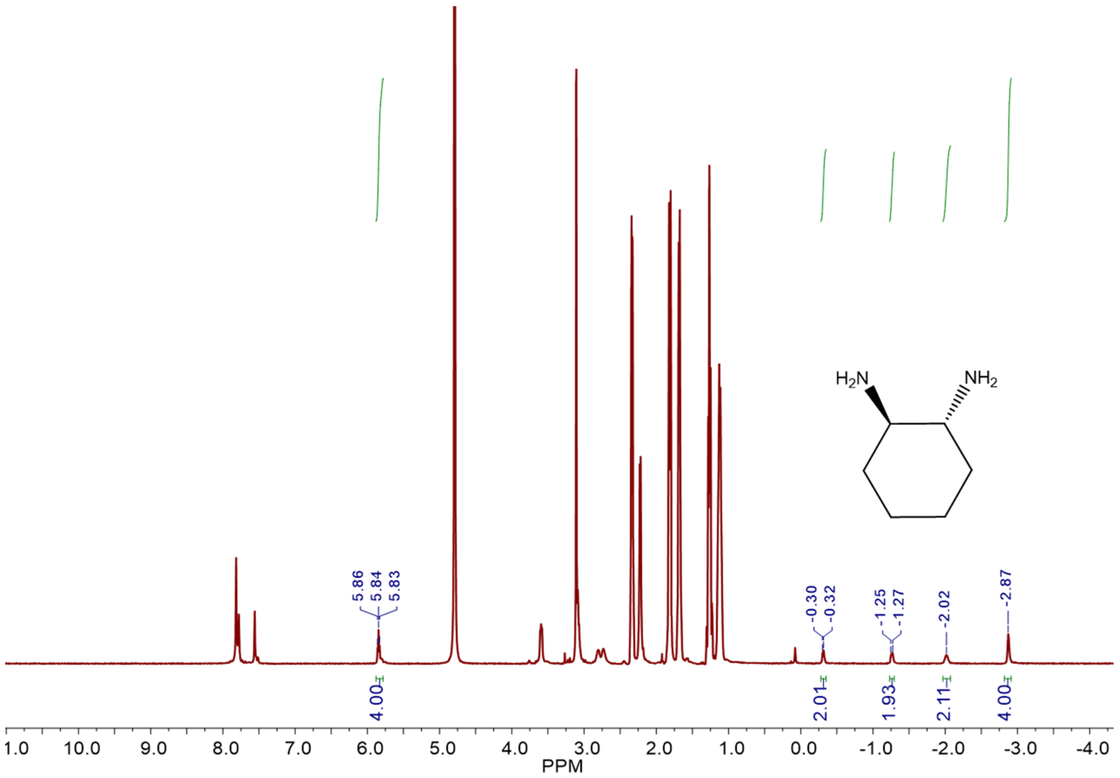

Fig. S23 ^{1}H NMR spectrum of the complex formed between cavitand **1** and excess of trans-1,2-diaminocyclohexane in D_2O

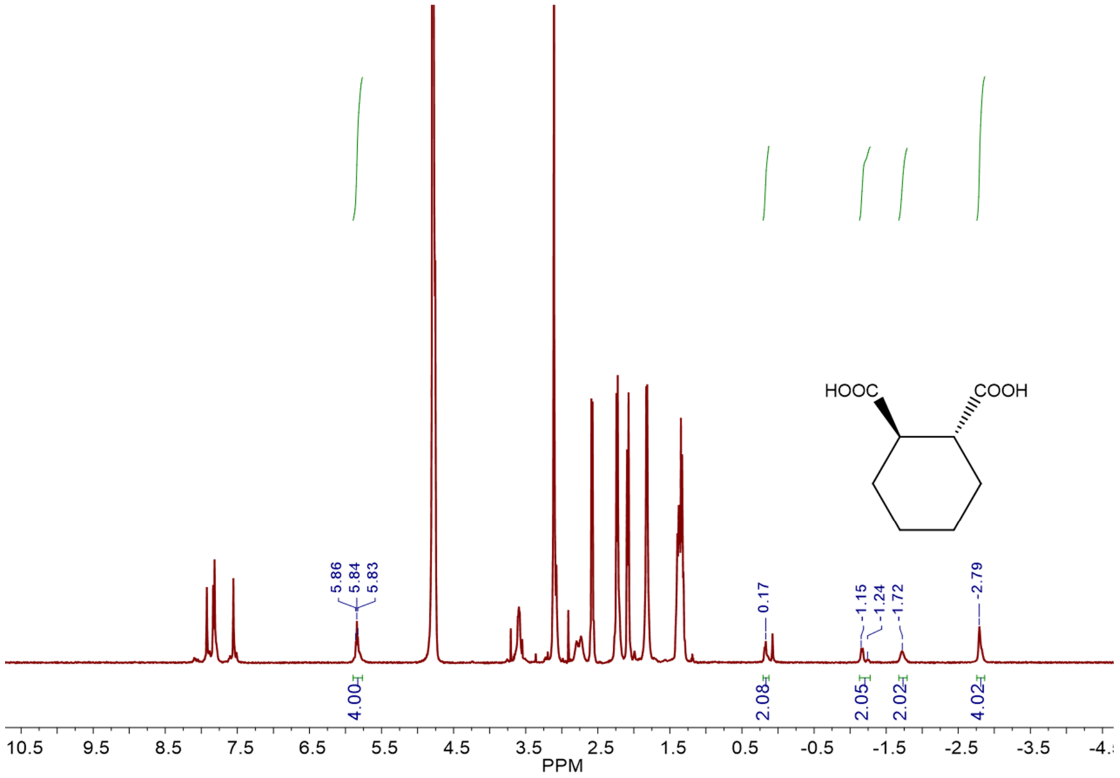

Fig. S24 ^{1}H NMR spectrum of the complex formed between cavitand **1** and excess of trans-1,2-Cyclohexanedicarboxylic acid in D_2O

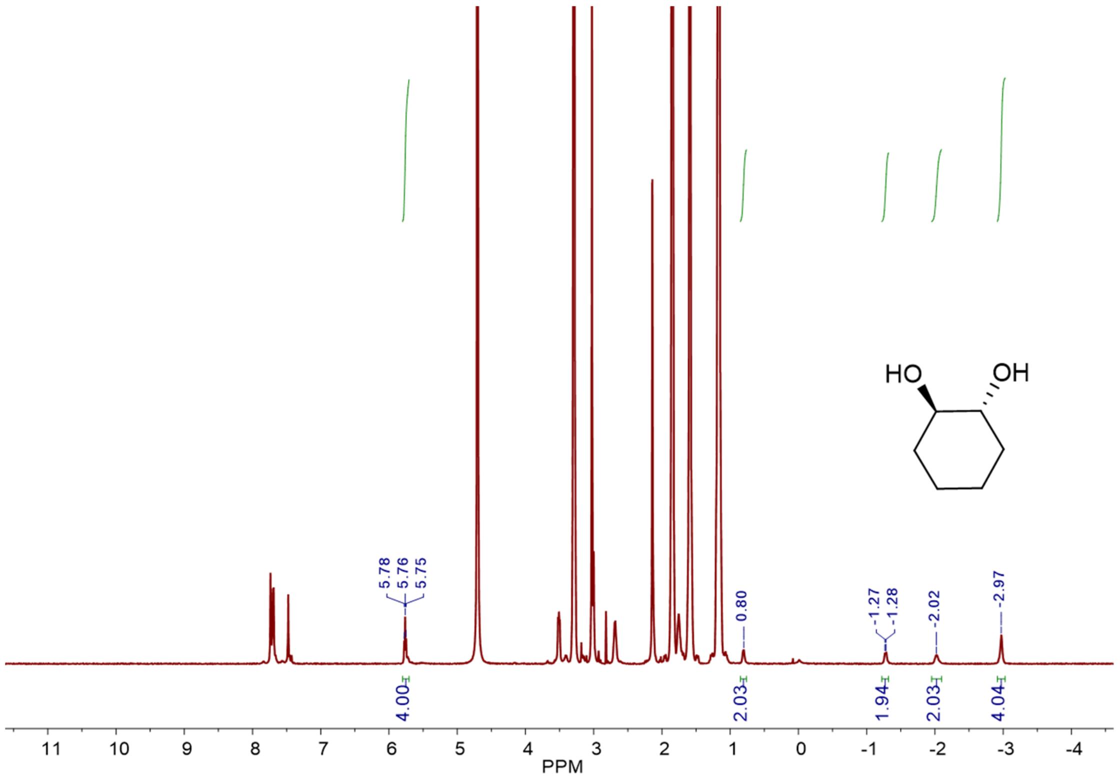

Fig. S25 ^{1}H NMR spectrum of the complex formed between cavitand **1** and excess of trans-1,2-Cyclohexanediol in D_2O

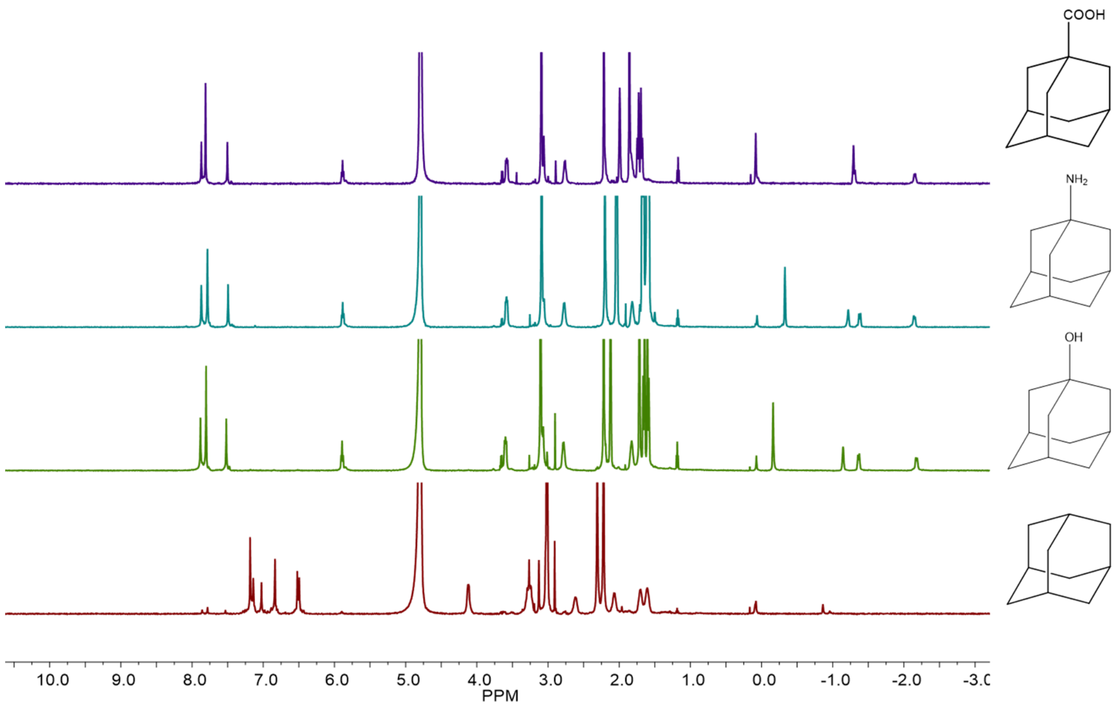

Fig. S26 ^{1}H NMR spectra of the complexes formed between cavitand **1**, 1mmol + from bottom to top, exc ess of admantane, 1-adamantanol, amantadine, 1-adamantanecarboxylic acid

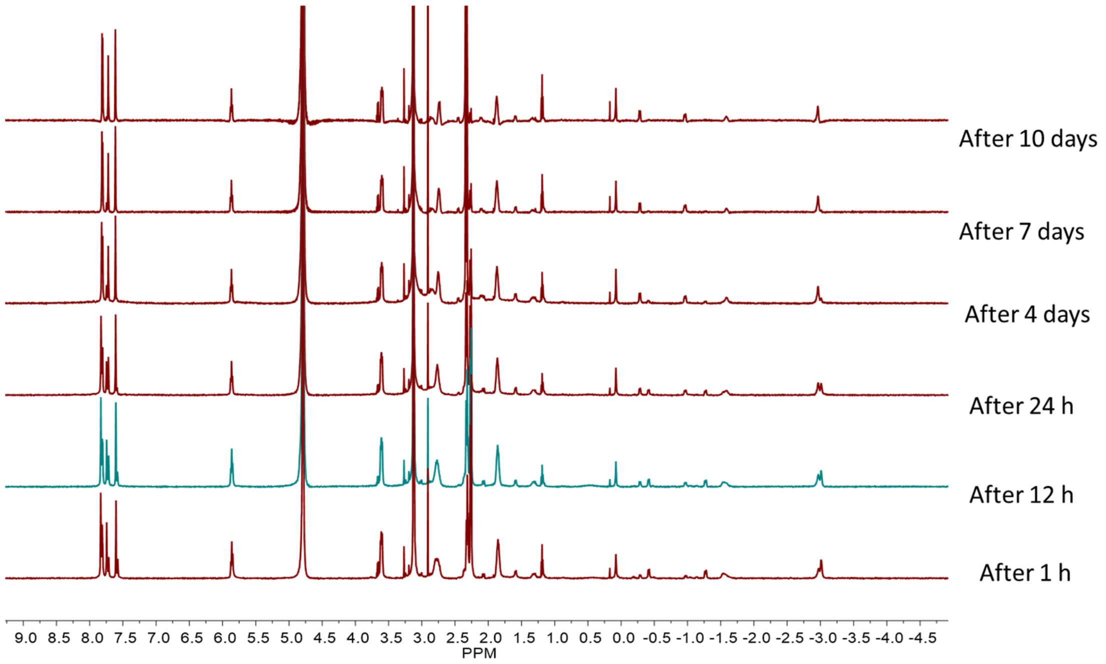

Fig. S27 Comparative ^1H NMR spectra of the host-guest complex formed between equimolar oxaliplatin and cavitand **1** in D_2O, after stand for from bottom to top 1h, 12 h, 24 h, 4 days ,7 days and 10 days
